# 

**Published:** 1824-12

**Authors:** 


### MONTHLY CATALOGUE OF MEDICAL BOOKS.

*** *It having been hinted to us that gentlemen, sending copies of their Works, prefer having their titles given at length, it is our intention, in future, to comply with this suggestion: but it is to be observed, that no books can be entered on this List except those sent to us for the purpose;—finding, in the lists hitherto transmitted, that the names of works have frequently been given as published, which have not appeared for weeks, or even months after.*

An Essay on Curvatures and Diseases of the Spine, including all the Forms of Spinal Distortion; to which the Fothergillian Gold Medal was awarded by the Medical Society of London, and presented at a special General Meeting, on the 3d of May, 1824; with some Additions. By R. W. BAMPFIELD, Esq. one of the Surgeons to the Royal Metropolitan Infirmary for Children; Fellow of the Medical Society of London; author of "an Essay on Hemeralopia, or Night-Blindness," of "Practical Treatises on Tropical and Scorbutic Dysentery," &c. —Pp. 387. London, 1824.

Nosographie Medicale, ou Elémens de Medecine Pratique, à l'usage des Elevés en Medecine et en Chirurgie, et de tous les Hommes de l'Art auxquelles la pratique ne permet pas de consulter un grand nombre d'Ouvrages. Par S. P. AUTHENAC, Docteur de la Faculté de Medecine de Paris, &c. &c. Tome premier.—Pp. 547. Paris, 1824.

De Primariis quibusdam in Medicamentorum Viribus recte Œstimandis, &c. Auctore CAROLO TRINKS, Med. et Chir. Doctor.—Pp. 26. Lipsiæ, 1823.

De Morbis quibusdam Pulmonum rarioribus Commentatio Pathologica-Anatomica. Auctore GULIELMUS MAYER, M.D.—Pp. 25, cum Tabula ænea. Lipsiæ, 1824.

Considerations sur les Convulsions qui attaquent les Femmes enceintes, lues a la sceance publique de la Maison d'Accouchment, le 25 Juin, 1823. Par M. CHAUSSIER, &c. &c.—Pp. 23. Paris, 1824.

Hints respecting the Improvement of the Literary and Scientific Education of Candidates for the Degree of Doctor of Medicine, in the University of Edinburgh. Humbly submitted to the consideration of the Patrons and Professors of that Institution. By a Graduate of King's College, Aberdeen.—Pp. 21. Edinb. 1824.

An Inquiry into the Nature, Causes, and Cure of Hydrothorax: illustrated by interesting Cases, and many living Examples of the Success of the Mode of Treatment recommended. By L. MACLEAN, M.D. &c.—Pp. 218. Sudbury, 1810.

Traité des Maladies du Cœur et des gros Vaisseaux. Par R. I. BERTIN, &c. &c. Redigé par J. BOUILLARD, M.D.—Avec six planches, pp. 464. à Paris, 1824.

L'Art de Prolonger la Vie de l'Homme; par C. F. HUFELAND, premier Medecin et Conseiller d'Etat de S. M. le Roi de Prusse, &c. &c. &c. Traduit de l'Allemand sur la seconde Edition, par A. J. L. JOURDEN, Docteur de Medecine de la Faculté de Paris, &c.—Pp. 436. Paris, 1824.

A Nosological Practice of Physic, embracing Physiology. By GEORGE PEARSON DAWSON, M.D.—Pp. 380. London, 1824.

Clinical Report on Dropsies, with Observations explanatory of their Pathology and Therapeutics; with an Appendix, on the Theory and Treatment of Organic Diseases in general. By ROBERT VENABLES, Bachelor in Medecine and Licentiate in Physic of the University of Oxford; Physician to the Henley Dispensary, and consulting Physician to the Poor-house.—Pp. 238. London, 1824.

---

### NOTICE TO CORRESPONDENTS.

*The Communications of* Mr. LIDDELL, Dr. VANDEBURGH, Dr. CRAWFORD, Dr. COLLINS, Mr. YEATMAN, Mr. BROWNE, Mr. BUSH, "A MEDICAL STUDENT," Dr. DRUG, Dr. RIDGWAY, Mr. WALLER, *and* Dr. WHYMPER, *have been received.*

*The Communication from Dublin we have taken the liberty of handing over to our respected Contemporary, to whom it more peculiarly belongs.*

